# Epilepsy‐associated SCN2A‐L1342P mutation drives network hyperexcitability and widespread transcriptomic changes in human cortical organoids

**DOI:** 10.1002/epi.70392

**Published:** 2026-07-30

**Authors:** Maria I. Olivero‐Acosta, Morgan Robinson, Zhefu Que, Zaiyang Zhang, Karen V. Salazar‐Salazar, Hope Elizabeth Harlow, Vinayak Shankar, Seoyeon Hong, Muhan Wang, Conrad M. Otterbacher, Hina Kadono, Manasi Halurkar, Harish Kothandaraman, Nadia Atallah Lanman, Trang Nguyen, Kyle Wettschurack, Benjamin Zirkle, Layan Yunis, Ningren Cui, Xiaoling Chen, Jingliang Zhang, Jiaxiang Wu, William C. Skarnes, Chongli Yuan, Feng Guo, Megan Abbott, Yang Yang

**Affiliations:** ^1^ Borch Department of Medicinal Chemistry and Molecular Pharmacology, College of Pharmacy Purdue University West Lafayette Indiana USA; ^2^ Purdue Institute for Integrative Neuroscience (PIIN) Purdue University West Lafayette Indiana USA; ^3^ Davidson School of Chemical Engineering Purdue University West Lafayette Indiana USA; ^4^ Purdue University Institute for Cancer Research Purdue University West Lafayette Indiana USA; ^5^ Department of Comparative Pathobiology Purdue University West Lafayette Indiana USA; ^6^ Cellular Engineering The Jackson Laboratory for Genomic Medicine Farmington Connecticut USA; ^7^ Department of Intelligent Systems Engineering Indiana University Bloomington Indiana USA; ^8^ Pediatric Neurology University of Colorado School of Medicine Aurora Colorado USA

**Keywords:** cortical organoids, L1342P, multi‐electrode array, *SCN2A*, voltage‐gated sodium channel Nav1.2

## Abstract

**Objective:**

*SCN2A* pathogenic mutations, such as the recurrent heterozygous Nav1.2‐L1342P, are monogenic causes of epilepsy. In this human‐induced pluripotent stem cell–derived model system, we aim to investigate the molecular and cellular mechanisms underlying *SCN2A*‐L1342P‐associated pathology.

**Methods:**

Using a human male induced pluripotent stem cell (iPSC) reference line (KOLF) carrying the Nav1.2‐L1342P mutation, we generated three‐dimensional (3D) cortical organoids for functional studies. Patch‐clamp, multi‐electrode array (MEA) recordings, immunocytochemistry, and RNA sequencing were used to characterize the disease phenotypes.

**Results:**

Nav1.2‐L1342P organoid neurons displayed increased intrinsic excitability and amplified excitatory post‐synaptic currents, which are consistent with an increase in excitatory synapse formation revealed by SYN1/PSD95 immunostaining. Moreover, elevated network firing activity, as demonstrated by MEA, indicates a pronounced network hyperexcitability. Transcriptomic profiling of organoids carrying the Nav1.2‐L1342P mutation further revealed significant alterations in synaptic, glutamatergic, developmental, and senescence/apoptotic pathways.

**Significance:**

Our findings demonstrate that the Nav1.2‐L1342P mutation drives a multifaceted disease phenotype, including network hyperexcitability and disruption of pathways related to neuronal and synaptic functions. These results advance our understanding of *SCN2A*‐related developmental and epileptic encephalopathy (DEE), laying a foundation for personalized interventions.

**Key points**

- Human neurons carrying epilepsy‐causing *SCN2A*‐L1342P display intrinsic hyperexcitability in cortical organoids.
- Synaptic transmission is enhanced in organoids carrying the *SCN2A*‐L1324P mutation, consistent with the presence of elevated excitatory synapses, which leads to increased network hyperexcitability.
- Transcriptomics analysis reveals that the *SCN2A*‐L1342P mutation causes significant differential changes in genes involved in forebrain development, synaptic and glutamatergic signaling pathways, and enhances cellular senescence and apoptosis.

## 1 INTRODUCTION

The *SCN2A* gene encodes the alpha subunit of the neuronal voltage‐gated type II sodium channel Nav1.2, which plays a key role in action potential (AP) initiation and propagation.^1^, ^2^ Mutations in *SCN2A*, particularly gain‐of‐function (GoF) variants, are a known cause of early infantile developmental epileptic encephalopathy (EIEE).^3^ Among these, the recurrent Nav1.2‐L1342P (p.Leu1342Pro) mutation has been identified in at least five patients worldwide.^4^, ^5^, ^6^, ^7^ Biophysical and cellular studies have shown that the L1342P variant mainly displays a GoF phenotype.^8^, ^9^ Clinically, affected individuals with the Nav1.2‐L1342P variant present with severe intellectual disability, hypsarrhythmia, infantile spasms, and epileptic spasms,^4^ often accompanied by transient choreatic movement disorders and hypersomnia.^6^ Reports of brain atrophy have also been documented in the majority of individuals with the Nav1.2‐L1324P variant.^4^, ^10^ Despite these findings, the molecular and cellular mechanisms by which the Nav1.2‐L1342P mutation disrupts neuronal function remain poorly understood.

To study the cellular and molecular mechanisms of the L1342P variant, cortical organoids, three‐dimensional (3D) self‐organizing aggregates derived from human‐induced pluripotent stem cells (hiPSCs), are emerging as advanced models that resemble features of the dorsal forebrain.^11^, ^12^, ^13^ Cortical organoids exhibit a neuroectoderm‐like epithelium that matures over time into cortical‐resembling neurons.^14^ At later developmental stages, a heterogeneous cellular composition emerges, comprising glutamatergic neurons with characteristics of both deep and superficial layers (e.g., T‐box brain transcription factor 1 (TBR1)‐positive neurons). These neurons form layer‐like structures, establish synaptic connections, and display electrical activity, making them physiologically relevant models for electrophysiological studies.^14^, ^15^

In the present study, we established a 3D cortical organoid model to further investigate the molecular, cellular, and network consequences of the epilepsy‐associated Nav1.2‐L1342P mutation. Electrophysiological analyses of our 3D model using patch‐clamp recordings revealed that Nav1.2‐L1342P neurons exhibit enhanced repetitive AP firing, increased intrinsic neuronal excitability, and elevated network‐level neuronal activity. This hyperactive profile is further supported by a pronounced increase in spontaneous excitatory post‐synaptic currents (sEPSCs) and a heightened number of excitatory synapses. In parallel, bulk RNA‐sequencing (RNA‐Seq) of cortical organoids with the Nav1.2‐L1342P mutation revealed significant dysregulation in synaptic, glutamatergic, neurodevelopment‐related, and apoptotic/senescent transcriptional pathways, suggesting broad alterations in gene expression. These findings indicate that the Nav1.2‐L1342P mutation not only drives electrophysiological changes but also induces widespread transcriptional disruptions, providing mechanistic insights into the seizures and abnormal brain structure observed in patients with this mutation.

## 2 RESULTS

### 2.1 The Nav1.2‐L1342P mutation enhances intrinsic excitability of hiPSC‐derived cortical organoid neurons

We previously demonstrated that two‐dimensional (2D) cortical neuron monolayers carrying the Nav1.2‐L1342P mutation exhibit mainly a GoF phenotype^8^ and increased glutamate release.^9^ However, 2D models lack 3D tissue architecture. Therefore, in this study, we utilized a cortical organoid model to investigate how the Nav1.2 L1342P mutation influences neuronal excitability at both single‐cell and network levels. Cortical organoids (Figure 1) were generated from hiPSCs using a dual‐SMAD inhibition medium protocol,^16^ supplemented with small molecules to induce cortical neuron fate (Figure 1A,B). On Day 30, we identified high proportions of neural progenitor cells positive for SRY‐box transcription factor‐2 (SOX2), which also had proliferative capacity, as seen by proliferation marker Ki67 (Figure 1C). On Day 120 of maturation, differentiated cortical organoids contain neuronal nuclear antigen‐positive (NEUN+) neurons (Figure 1D) and T‐box brain transcription factor 1 (TBR1+) cortical neurons (Figure 1E), which are arranged in a layer‐like fashion. Beta‐tubulin‐3 (TUJ1) was also found throughout the organoids (Figure 1E), demonstrating widespread neuronal architecture. These data provide evidence that the hiPSCs can be differentiated into cortical organoids.

**FIGURE 1:**
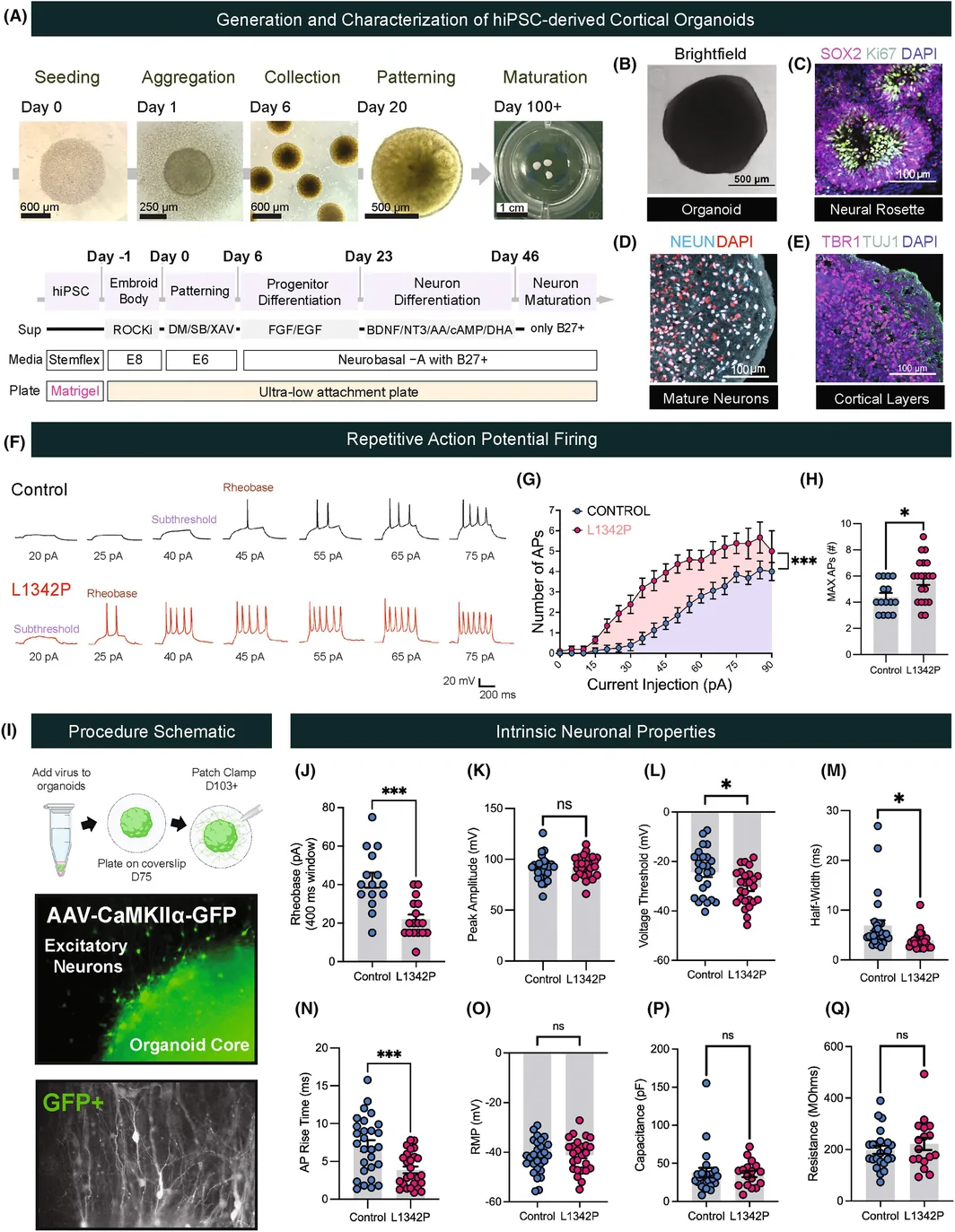
The Nav1.2‐L1342P mutation enhances the repetitive firing of neurons in hiPSC‐derived cortical organoids. (A) Experimental schematic for the generation of hiPSC‐derived cortical organoids. (B) Representative brightfield image of a 50‐day‐old cortical organoid. (C) Day 30 cortical organoid immunocytochemistry for neural progenitor marker SOX2 (magenta), proliferation marker Ki67 (green), and cell nuclei stain DAPI (blue). Neural rosettes contain neural progenitor cells (NPCs), radially organized in a central lumen resembling an embryonic neural tube, and appear as flower‐like patterns. (D) One hundred twenty‐day‐old cortical organoids express neuronal nuclear antigen marker (NEUN) (cyan) and 4′,6‐diamidino‐2‐phenylindole (DAPI) (red). (E) One hundred twenty‐day‐old cortical organoids express cortical marker T‐box brain transcription factor 1 (TBR1) (magenta), arranged in a layer‐like formation, along with neuronal marker beta tubulin‐3 (TUJ1) (green). (F) Preparation of hiPSC‐derived cortical organoids for patch‐clamp experiments. Organoids are transduced with an adeno‐associated virus (AAV) carrying a calcium/calmodulin‐dependent protein kinase II alpha (CaMKIIa)‐driven green fluorescent protein (GFP) to label excitatory neurons (green) and are subsequently plated onto coverslips. Neurites emanate from the cortical organoid, forming a monolayer and becoming available to patch after day 103 of organoid development. (G) Representative sustained action potential (AP) firings from Nav1.2‐L1342P neurons (red) or controls (blue). (H) AP number per epoch in response to graded inputs (0 to 90 pA, 400 ms). Data analyzed by repeated‐measures two‐way ANOVA (***p < 0.001). (I) The maximum number of action potential firings is increased in the Nav1.2‐L1342P cortical organoid neurons (Control: 4.4 ± 0.3 APs, *n* = 15 neurons, two clones across three differentiations; L1342P: 5.7 ± 0.4 APs, *n* = 20 neurons, three clones across five differentiations, *p = 0.0203, Mann–Whitney *U* test). (J) The rheobase was decreased in Nav1.2‐L1342P cortical organoid neurons (Control: 42.3 ± 3.9 pA, *n* = 15 neurons, two clones across three differentiations; L1342P: 22.1 ± 2.4 pA, *n* = 17 neurons, three clones across five differentiations; ***p = 0.0002, Welch's *t* test). (K) The peak amplitude was unchanged between conditions (Control: 90.5 ± 2.3 mV, *n* = 28 neurons, two clones across five differentiations; L1342P: 92.9 ± 2.1 mV, *n* = 26 neurons, three clones, across seven differentiations; ns, *p* = 0.4348, unpaired Student's *t* test). (L) The voltage threshold was reduced in Nav1.2‐L1342P cortical organoids (Control: −24.4 ± 1.9 mV, *n =* 25 neurons, two clones across five differentiations; L1342P: −30.5 ± 1.4 mV, *n* = 26 neurons, three clones, across seven differentiations; *p = 0.0127, unpaired Student's *t* test). (M) The half‐width was reduced in Nav1.2‐L1342P cortical organoids (Control: 6.90 ± 1.05 ms, *n* = 28 neurons, two clones across five differentiations; and L1342P: 3.94 ± 0.35 ms, *n* = 26 neurons, three clones, across seven differentiations; *p = 0.0128, unpaired Student's *t* test). (N) The rise time was decreased in Nav1.2‐L1342P cortical organoids (Control: 7.08 ± 0.73 ms, *n* = 28, two clones across five differentiations; L1342P: 3.88 ± 0.45 ms, *n* = 26 neurons, three clones, across seven differentiations; ***p = 0.0006, unpaired Student's *t* test). (O) The resting membrane potential (RMP) was unchanged between conditions (Control: −41.7 ± 1.3 mV, *n* = 28 neurons, two clones across five differentiations; L1342P: −41.2 ± 1.3 mV, *n* = 26 neurons, three clones, across seven differentiations; *p* = 0.7631, unpaired Student's *t‐test*) (P) The neuronal cell capacitance, reflecting the ability of the cell membrane to store charge, remained unchanged between groups (Control: 37.76 ± 6.29 pF, *n* = 23 neurons, two clones across five differentiations; L1342P: 36.01 ± 4.09 pF, *n* = 17 neurons, three clones across seven differentiations; ns, *p* = 0.8307, unpaired Student's *t* test). (Q) Cell resistance was also comparable between conditions (Control: 199.0 ± 15.74 MΩ, *n* = 23 neurons, two clones across five differentiations; L1342P: 220.0 ± 23.85 MΩ, *n* = 17 neurons, three clones across seven differentiations; ns, *p* = 0.4078, unpaired Student's *t* test). Data are reported as mean ± error (SEM). Each dot corresponds to one neuron.

To investigate how the Nav1.2‐L1342P mutation impacts both neuronal repetitive firing and the intrinsic properties of individual APs, we performed whole‐cell current‐clamp. Organoids were cultured for 75–80 days, then transduced with an adeno‐associated viral vector encoding green fluorescent protein under the control of the calcium/calmodulin‐dependent protein kinase II alpha promoter (AAV‐CaMKIIa‐GFP) to selectively label excitatory neurons. Transduced organoids were plated onto poly‐L‐ornithine (PLO)‐laminin‐coated glass coverslips, and maintained until 103–157 days of maturation before recordings (Figure 1I). Only GFP‐positive pyramidal‐shaped neurons were targeted for analysis.

Compared with controls, CaMKIIa+ Nav1.2‐L1342P neurons displayed a significant increase in repetitive firing during 400 ms current injections (from 0 to 90 pA, 5‐pA increments). From the 25 to 75 pA stimuli, mutants fired two to seven APs in response to increasing current steps (Figure 1F, top). In contrast, zero or at most four APs were elicited in control neurons (Figure 1F, bottom). Overall, the Nav1.2‐L1342P neurons fired significantly more APs than controls in the 0–90 pA increasing current injections (Figure 1G). Notably, the maximum number of AP firings that could be triggered was also significantly higher in Nav1.2‐L1342P neurons, showing a 29.5% increase over controls (Figure 1H), consistent with a hyperexcitable phenotype.

To further dissect intrinsic AP properties, cortical neurons were held at −65 mV and stimulated with 20 ms current steps (0–250 pA, 5 pA increment steps). Quantitatively, we found a reduction in the rheobase of the Nav1.2‐L1342P cortical organoid neurons compared to controls, indicating that less current is needed to elicit an AP in the mutant condition (Figure 1J). AP amplitude did not differ between control and Nav1.2‐L1342P neurons (Figure 1K). We further analyzed the voltage threshold of spiking, as sodium channel dysfunction is likely to influence this parameter. Our results revealed that Nav1.2‐L1342P neurons had a significantly lower voltage threshold, indicating a higher probability of AP firing at a more negative membrane potential (Figure 1L).

The Nav1.2‐L1342P neurons also increased the rate of AP firing. We found a statistically significant decrease in AP (spike) half‐width in Nav1.2‐L1342P cortical organoid neurons compared to controls, indicating a shorter AP duration (Figure 1M). In addition, the AP rise time was reduced in the Nav1.2‐L1342P neurons compared to controls, indicating a faster AP initiation (Figure 1N). Finally, we found that the basic membrane properties of our recorded excitatory neurons, including resting membrane potential (RMP) (Figure 1O), capacitance (Figure 1P) and input resistance (Figure 1Q), were unaffected in the neurons carrying the Nav1.2‐L1342P mutation.

### 2.2 Nav1.2‐L1342P cortical organoid neurons show enhanced excitatory synaptic transmission and elevated synapse formation compared to controls

In addition to increased intrinsic excitability, we assessed sEPSCs that mediate synaptic transmissions and contribute to network excitability.^17^ To this end, we evaluated sEPSCs in both wild‐type (WT) control cortical organoids (Figure 2A, top) and those carrying the Nav1.2‐L1342P variant (Figure 2A, bottom). Compared with controls, Nav1.2‐L1342P organoids exhibited significantly increased sEPSC frequency (Figure 2B) and amplitude (Figure 2C), reflecting more frequent and stronger synaptic events. Furthermore, the inter‐event interval was reduced in the mutants (Figure 2D), further confirming enhanced synaptic activity. Together, these results indicate that the Nav1.2‐L1342P variant strengthens excitatory transmission, consistent with our previous finding of increased glutamate release.^9^

**FIGURE 2:**
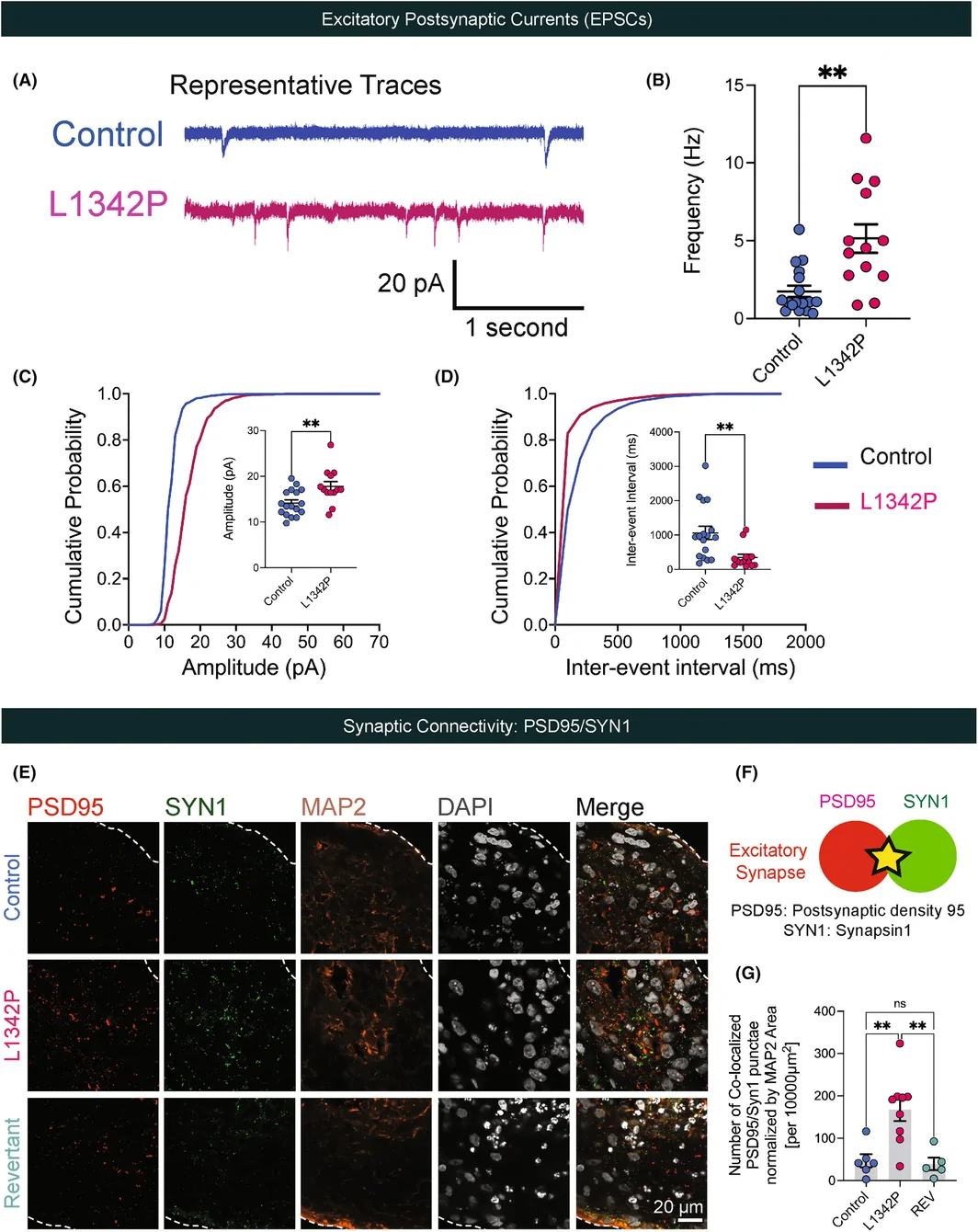
Nav1.2‐L1342P cortical organoid neurons display enhanced excitatory synaptic transmission and enhanced synapse formation compared to isogenic controls. (A) Representative spontaneous excitatory post‐synaptic current (sEPSCs) traces recorded from Control and Nav1.2‐L1342P cortical organoid neurons. (B) sEPSC frequency is increased in Nav1.2‐L1342P cortical organoid neurons (Control: 1.74 ± 0.36, *n =* 17 neurons, three clones across five differentiations; L1342P: 5.14 ± 0.91, *n* = 13 neurons, three clones across three differentiations; **p = 0.0021, Mann–Whitney *U* test). (C) The cumulative probability distribution of sEPSC amplitude, with quantification indicating an increase in amplitude within Nav1.2‐L1342P cortical organoids (Control: 14.2 ± 0.7 pA, *n* = 17 neurons, three clones across five differentiations; L1342P: 17.8 ± 1.0 pA, *n* = 13 neurons, three clones across three differentiations; **p = 0.0100, Mann–Whitney *U* test). (D) The cumulative probability distribution of sEPSC inter‐event interval, with quantification revealing decreased inter‐event intervals in Nav1.2‐L1342P cortical organoids (Control: 1059 ± 193 ms, *n* = 17 neurons, three clones across five differentiations; L1342P: 345.1 ± 94.3 ms, *n* = 13 neurons, three clones across three differentiations; **p = 0.0021, Mann–Whitney *U* test). (E) Enhanced synapse formation in Day 120 cortical organoids carrying the Nav1.2‐L1342P mutation. Postsynaptic density 95 PSD95 (magenta) and presynaptic Synapsin‐1 (green) puncta are enriched along the outer regions of the organoid, where neurons are predominantly localized. MAP2 (orange) outlines the neuronal somatodendritic processes and organoid boundaries, whereas DAPI (white) labels all cell nuclei. (F) Components of an excitatory synapse. (G) The number of co‐localized puncta (synapses) was increased in the Nav1.2‐L1342P Cortical organoids compared to controls and revertant lines (Control: 46.8 ± 15.6 punctae per 10000 μm^2^, *N* = 6 organoids, *n* = 36 images, two clones across three differentiations; L1342P: 168.0 ± 27.2, punctae per 10000 μm^2^ *N* = 9 organoids, *n* = 53 images, two clones across three differentiations; REV: 39.6 ± 14.7 punctae per 10000 μm^2^, *N* = 5 organoids, *n* = 25 images, one clone across two differentiations). Data are reported as mean ± error (SEM). One‐way ANOVA, *p < 0.05; **p < 0.01; ns, non significant.

Increased sEPSCs could result from either increased formation of excitatory synapses or enhanced spontaneous neuronal firing. To test this, we performed immunocytochemistry of cortical organoids (Figure 2E) against two excitatory synaptic proteins: Synapsin‐1 (SYN1), a presynaptic protein crucial for synaptogenesis and synaptic plasticity,^18^ and PSD95, the post‐synaptic density protein‐95 involved in the stabilization, recruitment, and trafficking of N‐methyl‐D‐aspartate receptors (NMDARs) and alpha‐amino‐3‐hydroxy‐5‐methyl‐4‐isoxazolepropionic acid receptors (AMPARs) to the post‐synaptic terminals^19^ (Figure 2F). Synapse density was quantified as SYN1/PSD95 co‐localizations (synapses) normalized to microtubule‐associated protein‐2 (MAP2) positive area. At Day 120, Nav1.2‐L1342P cortical organoids exhibited significantly increased co‐localization density of SYN1/PSD95 compared to WT control and an isogenic revertant (REV) control (Figure 2G), indicating enhanced excitatory synapse formation. These findings support our sEPSC data indicating enhanced synaptic connectivity.

### 2.3 Cortical organoids carrying the Nav1.2‐L1342P mutation display marked network hyperexcitability

Previous single‐cell patch‐clamp recordings revealed increased intrinsic excitability and sEPSCs in Nav1.2‐L1342P cortical organoid neurons. To determine whether these changes extend to network excitability, we performed multi‐electrode array (MEA) recordings, which enabled high‐throughput extracellular monitoring of spikes and field potentials across neuronal populations^20^ (Figure 3A). Organoids from three conditions:WT control, Nav1.2‐L1342P, and isogenic revertant P1342L,were plated on top of electrodes (Figure 3B).

**FIGURE 3:**
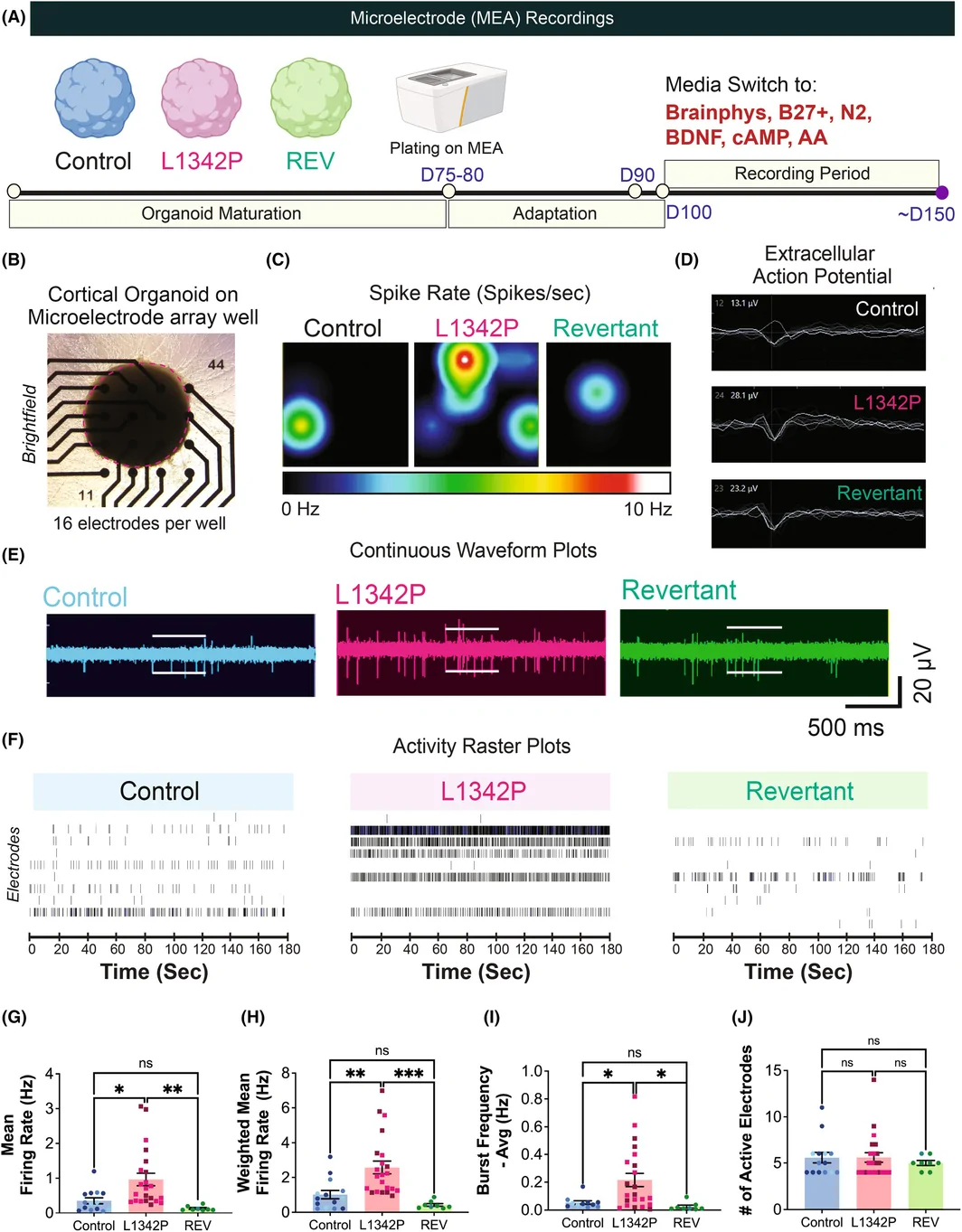
Multielectrode array (MEA) recordings reveal that cortical organoids carrying the Nav1.2‐L1342P mutation display marked network hyperexcitability. (A) Schematic description of organoids used in MEA experiments. (B) Brightfield image of a cortical organoid placed directly on top of MEA electrodes. (C) The firing of a population of neurons can be visualized in a heatmap view. Each colored circle represents an active electrode, with blue indicating a low firing frequency and yellow/red indicating a high firing frequency. Nav1.2‐L1342P cortical organoids exhibit higher spike rates compared to controls and revertants. (D) Representative extracellular action potential traces from an active electrode for each condition. (E) Continuous waveform plots showing spontaneous activity. Each spike indicates an event. Nav1.2‐L1342P cortical organoids display increased spontaneous activity values compared to controls and revertants. (F) Representative activity raster plots. Nav1.2‐L1342P cortical organoids had increased activity compared to controls and revertants. (G) The mean firing rate was increased in Nav1.2‐L1342P cortical organoids compared to controls and revertants (Control: 0.36 ± 0.08 Hz, *n* = 14 organoids, three clones across six differentiations; L1342P: 0.96 ± 0.18 Hz, *n* = 22 organoids, two clones, across six differentiations; REV: 0.14 ± 0.02 Hz, *n* = 8 organoids, two clones across two differentiations, one‐way ANOVA). (H) The weighted mean firing rate was increased in Nav1.2‐L1342P cortical organoids compared to controls and revertants (Control: 1.01 ± 0.24 Hz, *n* = 14 organoids, three clones across six differentiations; L1342P: 2.58 ± 0.37 Hz, *n* = 22 organoids, two clones across six differentiations; REV: 0.42 ± 0.08 Hz, *n* = 8 organoids, two clones across two differentiations, one‐way ANOVA). (I) The burst frequency of Nav1.2‐L1342P cortical organoids was increased compared to controls and revertants (Control: 0.054 ± 0.012 Hz, *n* = 11 organoids, three clones across six differentiations; L1342P: 0.216 ± 0.048 Hz, *n* = 22 organoids, two clones across six differentiations; REV: 0.025 ± 0.010 Hz, *n* = 8 organoids, two clones across two differentiations, one‐way ANOVA). (J) The number of active electrodes remained unchanged across sample groups. (Control: 5.5 ± 0.5 electrodes, *n* = 14 organoids, three clones across six differentiations; L1342P: 5.6 ± 0.5 electrodes, *n* = 22 organoids, two clones across six differentiations; REV: 5.0 ± 0.3 electrodes, *n* = 8 organoids, two clones across two differentiations, one‐way ANOVA). Data are reported as mean ± error (SEM). One‐way ANOVA, * *p* < 0.05; **p < 0.01; ***p < 0.001.

Nav1.2‐L1342P cortical organoids exhibited significantly higher neuronal network activity compared to controls and the isogenic revertant line, as seen in heatmaps (Figure 3C), extracellular AP traces (Figure 3D), continuous waveform plots (Figure 3E), and raster plots (Figure 3F). Quantitatively, MEA recordings revealed that the mean firing rate (MFR; number of spikes per second) of Nav1.2‐L1342P cortical organoids, was substantially elevated compared to controls and revertant cultures (Figure 3G), and the same trend was evident in the weighted mean firing frequency (WMFF; average firing rate normalized to the number of active electrodes), which was also elevated (Figure 3H). Bursting frequency, a seizure‐related firing pattern of neuronal cultures, was also significantly increased in Nav1.2‐L1342P cultures (Figure 3I). Notably, the number of active electrodes remained unchanged across conditions (Figure 3J). These findings suggest that the L1342P mutation enhances excitability at the neuronal network level in organoids.

### 2.4 Bulk RNA‐sequencing analysis of hiPSC‐derived cortical organoids reveals dysregulation of synaptic, glutamatergic, neurodevelopmental and apoptotic pathways

Following the functional characterization of Nav1.2‐L1342P cortical organoids, which revealed hyperexcitability, we investigated the molecular mechanisms underlying this phenotype using Bulk RNA sequencing. Six samples per condition were collected from at least two differentiations (Figure 4A). The sample similarity matrix (Figure 4B) and 3D Principal Component Analysis (PCA) plot also show a clear separation between mutant Nav1.2‐L1342P (pink) and WT control (blue) samples along PC1, PC2, and PC3 (Figure 4C). Our analysis identified a total of 5701 globally differentially expressed genes (DEGs) (false discovery rate [FDR] <0.05), including 2816 downregulated and 2354 upregulated genes (Figure 4D). The top 40 DEGs comprised 10 downregulated and 30 upregulated genes (Figure 4E).

**FIGURE 4:**
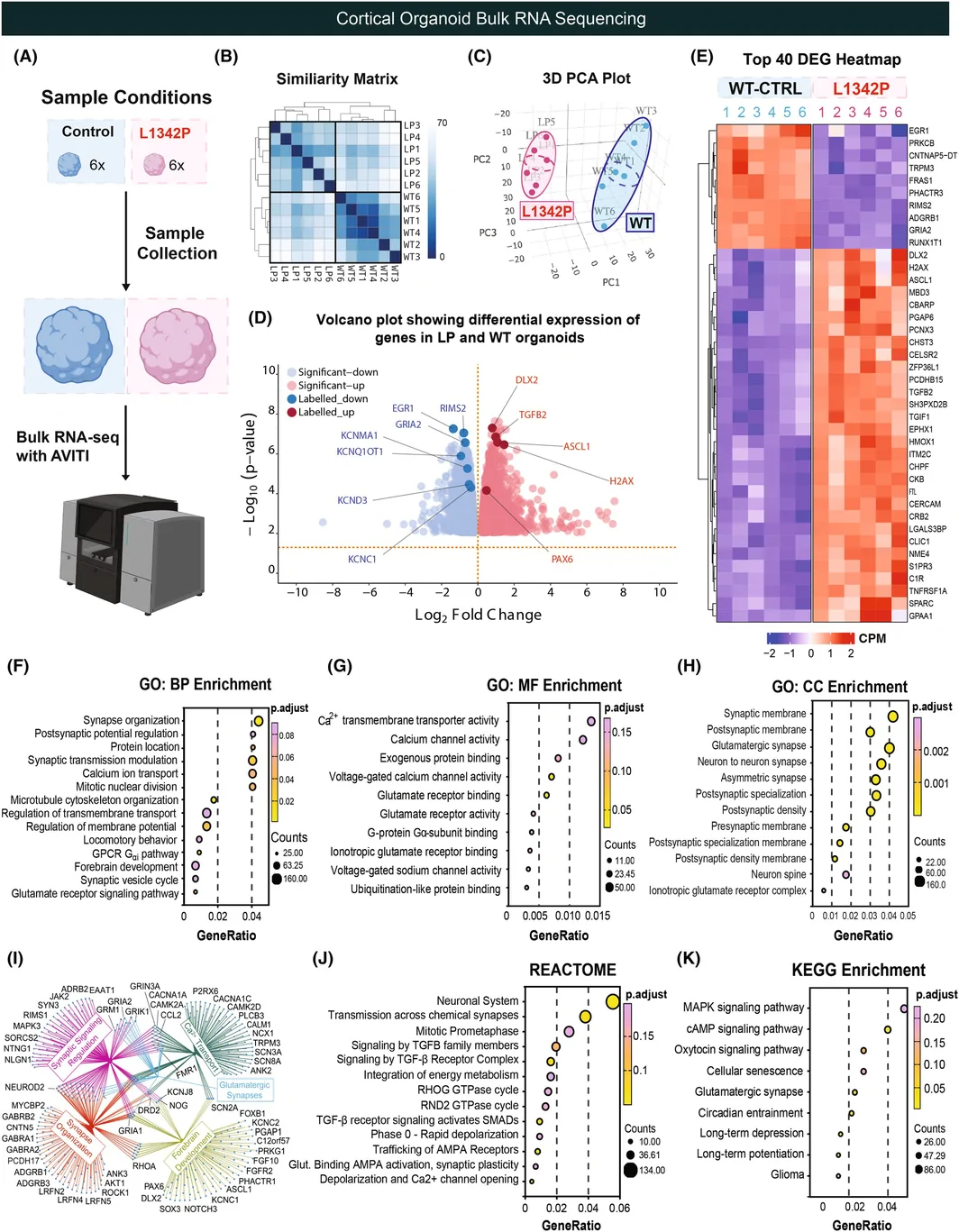
RNA sequencing analysis of hiPSC‐derived cortical organoids reveals impairments in synaptic, glutamatergic, forebrain developmental and apoptotic pathways. (A) Bulk RNA‐seq experimental conditions. Control organoids (*n* = 6) were derived from three independent differentiations and included KOLF2.1 J‐2 WT lines (*n* = 6; Day 120–167: one at Day 120, one at Day 154, three at Day 156, and one at Day 167) and one KOLF‐A03 WT organoid (Day 154). Nav1.2‐L1342P organoids (*n* = 6) were generated from two independent differentiations and comprised A12 HET organoids (*n* = 4, Day 131) and two E09 HET (*n* = 2; Day 154). All samples were on the KOLF2.1 J background and sequenced using the AVITI system. (B) Hierarchical clustering of cortical organoid sample replicates. Darker blue indicates higher similarity. (C) Principal component analysis (PCA) plot illustrating the clustering of replicates based on their variance in two dimensions. (D) Volcano plot highlighting significantly differentially expressed genes (DEGs, FDR <0.05) in Nav1.2‐L1342P hiPSC‐derived cortical organoids compared to controls. Notable downregulated genes include *SCN2A* and potassium channel–related genes. In contrast, upregulated genes include: *DLX2* (Distal‐Less Homeobox 2), *H2AX* (histone family member X), *PAX6* (Paired Box‐6), and *ASCL1* (Achaete‐scute family bHLH transcription factor 1), which are important in neurodevelopment, neuronal function, and cellular function. (E) DEG Heatmap showing the top 50 down (blue) and upregulated (red) genes across conditions. (F) Gene Ontology (GO) analysis of biological processes (BP) reveals changes in synapse organization, microtubule‐cytoskeleton arrangements, glutamate receptor signaling, and forebrain development (top). (G) GO molecular function (MF) analysis reveals enrichment in ion channel activity and alterations in the glutamatergic pathway. (H) GO cellular component (CC) identifies synaptic pathway alterations in the Nav1.2‐L1342P cortical organoids. (I) Network analysis reveals numerous globally differentially expressed genes (DEGs) involved in synaptic signal regulation (magenta), calcium transport (dark green), synapse organization (orange), and forebrain development (lime). The colored edges connecting genes represent functional relationships or interactions within each cluster, highlighting how these DEGs collectively contribute to altered neuronal physiology. Synaptic signaling regulation genes (magenta), such as *GRIA2*, *GRIN3A*, and *JAK2*, modulate synaptic activity and neurotransmission. Calcium transport genes (dark green), including *CAMK2A* and *CACNA1A*, regulate calcium signaling. Synapse organization genes (orange), such as *ANK3*, *GABRA2*, and *GRIA1*, contribute to the structural organization and maintenance of neural connections. Forebrain development genes (lime) include key transcription factors and developmental regulators such as *SOX3*, *PAX6*, and *NOTCH3*, which are important in cortical development. (J) REACTOME pathway analysis reveals disruptions in cell division, metabolism, AMPA (α‐amino‐3‐hydroxy‐5‐methyl‐4‐isoxazolepropionic acid) receptor trafficking, and neuronal synapses. (K) KEGG pathway enrichment analysis reveals alterations in MAPK and cAMP signaling pathways, as well as glutamatergic synapses. The circle size represents the number of DEGs, ranked by p.adjust values, whereas the GeneRatio represents the proportion of DEGs associated with a particular pathway relative to the total DEG set.

To investigate the biological processes affected by the Nav1.2‐L1342P organoids, we performed functional enrichment analysis of differentially expressed genes using clusterProfiler (Figure 4F–K). GO Biological Process analysis revealed disruptions in synapse organization, microtubule‐cytoskeleton dynamics, glutamate receptor signaling, cell division, and forebrain development (Figure 4F). Key developmental genes, including *DLX2*, *ASCL1*, *H2AX*, *SCN2A*, *POU3F2*, *CHD5*, *SLC4A10*, and *PAX6* were dysregulated (Figure 4D,E,I), further suggesting the role of Nav1.2 in development. Notably, *PAX6* (logFC: 0.4718, *p*‐value: 6.876E‐05), a marker of neural progenitor cells; *H2AX* (logFC: 1.446, *p*‐value: 3.425E‐07), a key regulator of mitotic prometaphase; and *ASCL1* (logFC: 1.082, *p*‐value: 2.631E‐07), a master regulator of neurogenesis, were significantly upregulated. Of interest, some potassium channel‐related genes, for example (*KCNC1*, *KCNC2*), were differentially downregulated within this GO network.

Gene Ontology (GO) Molecular Function analysis revealed perturbations in calcium and sodium channel activity and glutamatergic signaling, indicating dysregulation of cellular excitability, which relates to the hyperexcitability and sEPSC data (Figure 4G). GO Cellular Component analysis, which describes subcellular structures and macromolecular complexes,^21^ also identified significant alterations in synaptic pathways (Figure 4H). Network pathway analysis grouped DEGs into four main functional clusters (Figure 4I).

REACTOME pathway analysis revealed disruptions in cell division, metabolism, AMPA receptor trafficking, and neuronal synapse function (Figure 4J). Of interest, KEGG pathway analysis revealed dysregulation in *MAPK* and cyclic AMP (cAMP) signaling, as well as glutamatergic synapses and cellular senescence (Figure 4K). In our transcriptomics analysis, the KEGG senescence pathway was found to be significantly enriched in L1342P organoids, with the upregulation of many genes associated with cellular senescence and autophagy. For example, among the top 10 upregulated genes were *H2AX* (logFC: 1.446, *p*‐value: 3.425E‐07), which is both a progenitor marker and a double‐stranded DNA break marker; and *NME4*, a nucleoside diphosphate kinase involved in early stages of mitophagy and autophagy^22^ (logFC: 0.918, *p‐*value: 5.626E‐08). *GPX1*, one of the top 100 DEGs, was also upregulated and encodes a glutathione peroxidase that scavenges reactive oxygen species during oxidative stress, particularly in status epilepticus.^23^ Apoptosis‐related genes significantly increased, including *CASP1* (logFC: 1.430; *p‐*value: 0.00014), an early apoptotic marker. Several neuroimmune‐related genes linked to autophagy and phagocytosis in epilepsy were also prominently upregulated, including *CXCL3* (logFC: 1.876, *p*‐value: 0.0023), *CCL2* (logFC: 2.271, *p*‐value: 0.0021), and *C1R* (logFC: 1.167, *p*‐value: 2.483E‐08).

Consistent with the enrichment of stress‐related pathways in the RNA‐seq dataset, we performed immunostaining for cleaved caspase‐3 (CC3), a canonical marker for apoptosis. The proportion of CC3 + cells was significantly increased in cortical organoids carrying the Nav1.2‐L1342P mutation (Figure S2). These findings validate our transcriptomic results, indicating that L1342P organoids may be undergoing enhanced cellular stress and apoptosis, a feature that may be linked to the reported cerebral atrophy phenotypes observed in patients. Together, our findings suggest that the Nav1.2‐L1342P mutation impacts neuronal development and function, further supporting its role in neurodevelopmental pathophysiology.

## 3 DISCUSSION

This study demonstrates that the recurring Nav1.2‐L1342P mutation has a profound impact on neuronal excitability and overall gene expression profile. In this 3D system, we observed a hyperexcitability phenotype characterized by increased repetitive neuronal AP firing, intrinsic excitability, network neuronal firing, and sEPSCs. Immunocytochemical analyses revealed an increased number of glutamatergic synapses, offering a cellular correlate for the observed network activity. Along with increased synapses, RNA sequencing of Nav1.2‐L1342P cortical organoids revealed significant dysregulation of genes associated with synaptic signaling, glutamatergic neurotransmission, neurodevelopment, and cellular apoptosis/senescence, further supporting the role of the Nav1.2‐L1342P mutation in epilepsy disease manifestation.

*SCN2A*‐L1342P affects multiple patients, including five reported in the literature.^4^, ^5^, ^6^, ^7^ Recently, a sixth male patient, born in 2024, now 2‐years‐old, was identified with the Nav1.2‐L1342P variant. Parents provided verbal consent to be included in this study and for de‐identified patient information to be reported. The patient's first symptoms were difficulty eating and hypotonia at 2 months of age, prompting an evaluation with magnetic resonance imaging (MRI), electroencephalography (EEG) (normal at this time), and genetic testing. *SCN2A* variant L1342P was discovered at 5 months of age, when the patient presented with tonic seizures and then subsequently epileptic spasms. EEG at 6 months of age was a BASED (Burden of Amplitudes and Epileptiform Discharges) score of 4, qualifying as hypsarrhythmia with evidence of epileptic spasms on EEG, overall meeting criteria for infantile epileptic spasms syndrome. Seizure control has continued to be difficult despite many medication trials, including prednisolone, adrenocorticotropic hormone (ACTH), vigabatrin, ketogenic diet, clobazam, and lacosamide. Currently, the patient is maintained on oxcarbazepine, rufinamide, and Epidiolex, with the best seizure control occurring with the addition of oxcarbazepine. Comorbidities included dystonia, chorea, hypotonia, neuro‐irritability, disordered sleep, feeding intolerance requiring a gastrostomy tube, and sensory hypersensitivity. The patient has required frequent hospitalizations due to infections, seizures, and neuro‐irritability, all of which often cause respiratory complications. Notably, although this patient presented with seizures at 5 months of age, slightly past the conventional age thought to represent typical GoF patients, his best seizure responses have been to sodium channel blocking agents, namely oxcarbazepine, suggesting a GoF feature of this mutation. This additional patient report corroborates the recurrent nature of the L1342P variant and suggests that this site may be a hotspot for mutation.

Brain organoid studies are informative for understanding disease mechanisms; most study designs typically use patient vs healthy donors or isogenic WT and mutant lines as the standard practice in the field. However, to further enhance the rigor of our study, we also included an additional revertant control in a few key experiments. The inclusion of isogenic revertant lines provides important validation that the observed neuronal hyperexcitability and increased synaptic density phenotypes are attributable to the Nav1.2‐L1342P variant. However, revertant controls were not used in all experiments.

Guided differentiation into 2D and 3D cortical neuronal models takes advantage of dual‐SMAD inhibition. However, the two differentiation formats differ in their methodology, including the specific small molecules used, their duration of use, the cellular environment (monolayer vs suspension), tissue organization, and the overall culture duration. To determine whether *SCN2A*‐L1342P‐associated phenotypes are conserved across model systems, we compared functional and network properties of our 3D cortical organoids with those of our previous 2D neuronal model.^8^, ^9^ In our previous work, we observed a leftward shift in the activation curve, causeing channels to open at lower input voltages, leading to increased susceptibility of AP firing.^8^ This is consistent with previous studies in HEK293T cells expressing recombinant Nav1.2 mutant channels, which classified the variant as exhibiting mixed but net GoF.^24^ Consistent with this biophysical mechanism, both of our cortical models (2D neuronal monolayers and 3D cortical organoids) displayed enhanced intrinsic excitability. Nav1.2‐L1342P neurons exhibited increased repetitive AP firing patterns and reduced rheobase, although the magnitude of the effect differed between models. In both systems, the voltage threshold was lower in *SCN2A*‐L1342P HETs, but the resting membrane potential remained unchanged. We also observed an increase in the peak AP amplitude in L1342P neurons in the 2D model but not in the 3D organoids. AP half‐width was unchanged in the 2D system but was reduced in the L1342P organoids. In the microelectrode array recordings, both systems showed increased weighted mean firing rate and elevated burst frequency in the mutant condition. Overall, in both 2D and 3D systems, the models exhibit convergent overall phenotypes of increased intrinsic excitability and heightened network activity in neurons carrying the Nav1.2‐L1342P variant. This cross‐platform consistency demonstrates that the mutation‐associated phenotype is robust and preserved across distinct in vitro model systems.

*SCN2A*‐L1342P is largely considered a GoF variant that results in neuronal hyperexcitability, a feature shared across multiple pathogenic *SCN2A* GoF mutations. Previous voltage‐clamp recordings of *SCN2A*‐L1342P mutation reveal a hyperpolarizing shift in sodium current activation and inactivation, along with a net increase in sodium current density, suggesting predominantly GoF features.^8^, ^24^ In addition to L1342P, two other GoF *SCN2A* mutations (*SCN2A*‐R1882Q and *SCN2A*‐E1803G) have been studied with an iPSC‐derived 2D neuron model. In contrast, the *SCN2A*‐R1882Q variant exhibits a depolarizing shift in the inactivation curve and increased persistent current, whereas *SCN2A*‐E1803G shows a reduced inactivation slope and increased persistent current, both showing clear GoF features. Regardless of the biophysical differences at the channel level, all three variants lead to neuronal hyperexcitability phenotypes and seizures.^24^, ^25^ In the present brain organoid work, transcriptomic profiling of Nav1.2‐L1342P cortical organoids revealed broad alterations in pathways related to neurodevelopment and autophagy‐senescence. Similar transcriptomic signatures have been reported in 2D iPSC‐derived neurons carrying *SCN2A* R1882Q and E1803G, which exhibited upregulation of neurodevelopmental regulator genes (e.g., *NEUROD6* and *FOXG1*) alongside dysregulation of extracellular matrix organization, cell adhesion, and proliferative pathways.^26^, ^27^ KEGG enrichment analysis in Nav1.2‐L1342P organoids identified activation of autophagy and senescence‐related processes, including increased expression of pro‐apoptotic and caspase‐associated genes, paralleling caspase‐related alterations observed in the 2D neuron model carrying the R1882Q mutation.^26^ In addition, oxidative stress‐related genes, such as *NME4* and *GPX1*, were also upregulated in our Nav1.2‐L1342P organoids, aligning with findings from the *SCN2A*‐E1803G model.^27^ This suggests that transcriptomic signatures in different *SCN2A* GoF mutations, to a degree, showed a level of convergence. Of note, such stress‐related transcriptional changes within disease organoid models may influence neuronal maturation and network function. The degree to which activation of cellular stress programs influences intrinsic and network‐level activity remains to be fully described. Further analysis of additional *SCN2A* variants across developmental timepoints may be necessary to establish the role of cellular stress pathways in shaping disease phenotypes in *SCN2A*.

In this study, we focused on the functional properties of the excitatory neurons, in particular CaMKIIa + cells, as they predominantly express *SCN2A* and are the dominant cell type in guided cortical organoids.^14^ Although transcriptomic data suggest potential alterations in neurodevelopmental markers, synaptic, and excitatory/inhibitory gene expression, bulk RNA‐seq reflects the transcriptional landscape across the entire organoid. This limitation is further compounded by spatial heterogeneity within organoids, with more mature excitatory neurons preferentially localized in peripheral regions of the cortical organoid. It is important to note that our electrophysiological measurements primarily captured the properties of these peripheral excitatory neurons. The co‐localization of synaptic markers SYN1 and PSD95 was also performed on the peripheral regions, aligning structural and functional assessments with the populations examined in our electrophysiological recordings. Thus, it is not surprising that certain global transcriptomic changes observed in the bulk RNA‐seq dataset may not directly parallel the electrophysiological results.

In summary, we have established a preclinical in vitro hiPSC‐derived cortical organoid model to investigate the effects of the L1342P mutation. This epilepsy‐associated *SCN2A* mutation causes developmental and epileptic encephalopathy and abnormal brain structure in human patients. Our findings validate the utility of hiPSC‐derived technologies for studying *SCN2A*‐related pathology, revealing that the GoF L1342P variant influences complex molecular and cellular mechanisms beyond abnormal hyperexcitability. This highlights the multifaceted impact of *SCN2A* variants and their capacity to disrupt cellular processes as additional disease etiologies. Together, our study represents an advancement in using cortical organoids to model *SCN2A* mutations and provides a platform for testing novel interventions in human cell‐based preclinical models.

## 4 METHODS

### 4.1 Generation of cortical organoids from hiPSCs

The hiPSC colonies were maintained as described previously.^8^, ^9^ In this study, we used previously characterized KOLF2‐C1 hiPSCs derived from a male donor, including WT (control) and CRISPR/Cas9‐engineered Nav1.2‐L1342P lines. All lines used in this study were derived from a common parental KOLF2.1 J background and are therefore isogenic, differing at the *SCN2A* locus (WT‐Control, heterozygous L1342P, and P1342L revertant‐corrected line). To confirm that the observed phenotypic differences were specifically attributable to the L1342P mutation, CRISPR/Cas9 gene‐corrected P1342L revertant lines were included in selected experiments, including MEA recordings and synaptic co‐localization analyses. Control lines included KOLF2.1 WT, A03 WT, and B11 WT. The mutant Nav1.2‐L1342P lines included A12 HET, A10 HET, and E09 HET. Isogenic revertant lines were D08 REV and A07 REV. All clone identifiers are specified in the corresponding figure legends to ensure clarity and reproducibility. The hiPSC lines were screened for off‐target insertions and deletions at genomic loci *SCN1A*, *SCN3A*, and *SCN8A* to ensure no unintended editing. No copy number variations (CNVs) were detected after genome editing. A pluripotency panel was also performed to validate iPSC quality (Figure S1).

Cortical organoids were generated using the protocol developed by the Pașca laboratory at Stanford.^16^ Briefly, hiPSCs were aggregated at a cell density of 100 cells/μL in 100 μL of Essential 8 medium in 96‐well round‐bottom ultra‐low‐attachment plates. The plates were centrifuged at 100 × *g* for 3 min to promote embryoid bodies (EBs) formation. EBs were maintained in Essential‐6 medium for the first 6 days, supplemented with 2.5 μM dorsomorphin, 10 μM SB‐4321542, and 1.25 μM XAV‐939. On Day 6, EBs were transferred to 6‐well plates and maintained in Neurobasal‐A medium with Glutamax, 1% Pen/Strep, and B27‐A, along with 20 μg/mL FGF2 and 20 μg/mL EGF. Medium changes were performed daily for 17 days, and then every 2 days until Day 23. From Day 23 to 46, the Neurobasal was supplemented with 20 ng/mL BDNF, 20 ng/ mL NT3, 50 μM cAMP, 10 μM DHA, and 200 μM ascorbic acid. From Day 46 onwards, organoids were maintained in maturation media composed of Neurobasal‐A basal medium supplemented with only B27 +, with medium changes every 4–5 days.

### 4.2 Electrophysiology of hiPSC‐derived cortical organoid neurons

To select neurons for electrophysiological recordings, we targeted excitatory neuronal populations expressing either Control or Nav1.2‐L1342P mutant channels, transduced with pAAV‐CaMKIIa‐GFP. For viral labeling, 2–3 cortical organoids at days 75–80 were incubated with 1 μL of pAAV‐CaMKIIa‐GFP (1×10^13^ vg/mL) in 200 μL of maturation media in Eppendorf tubes overnight at 37°C, 5% CO_2_. The following day, 800 μL of maturation media was added, and organoids were incubated for an additional 24 h.^28^

Following transduction, organoids were plated onto glass coverslips (Neuvitro, GG‐12‐1.5‐Pre) coated with poly‐L‐ornithine (PLO) and laminin. Coverslips were coated overnight at room temperature with a 1:5 dilution of 0.01% PLO in phosphate‐buffered saline (PBS), washed three times with PBS, and subsequently incubated with 10 μg/mL mouse laminin in DMEM/F12 for 2 h at 37°C.^9^ Organoids were positioned at the center of each coverslip. Successful attachment was assessed by the emergence of visible neurites extending from the organoid core, forming a cohesive neuronal monolayer, as monitored by phase‐contrast microscopy. To minimize variability across preparations, the size, morphology, and positioning of organoids were monitored, and only organoids with comparable characteristics were selected for analysis. All neurons were patched between Days 103–157, corresponding to late‐stage organoid development, and confirmed to be CaMKIIa positive, based on GFP fluorescence.

After 1 week, coverslip‐attached cortical organoids were transitioned to an electrophysiology‐supporting medium consisting of BrainPhys supplemented with B27 + and N2 supplements, 20 ng/mL BDNF, 50 μM cAMP, and 200 μM AA. Half medium changes were performed every 3–4 days, and cultures were maintained until reaching maturity (~120–160 days).

For current‐clamp recordings of APs, the internal solution contained (in mM): 122 KMeSO_4_, 4 KCl, 2 MgCl_2_, 0.2 EGTA, 10 HEPES, 4 Na_2_ATP, 0.3 tris‐GTP, and 14 tris‐phosphocreatine, adjusted to pH 7.25 with KOH and to an osmolarity of 295–305 mOsm.^29^ Whole‐cell current‐clamp signals were recorded using an Axon MultiClamp 700B amplifier (Molecular Devices), low‐pass filtered at 2 kHz, and digitized at 10 kHz with Axon Digidata 1550B plus HumSilencer A/D converter (Molecular Devices). AP peak amplitude, rise time, RMP, and half‐width were quantified from single action potential curves generated with 20 ms current injection using Clampfit 11.3.^29^ The AP threshold was defined as the membrane potential at which dV/dT exceeded 8 mV/ms during the upstroke and was calculated using Origin (Origin, version 9.7).^8^ For generating the AP firing vs current plot, a 400 ms current injection window was used to support repetitive firing with current steps from −20 pA to +90 pA in increments of 5 pA. Rheobase was defined as the minimum 400 ms current injection required to elicit ≥1 action potential. Individual cortical organoid neurons included in our patch‐clamp analyses met standard electrophysiological quality‐control criteria (e.g., stable resting membrane potential, low leak current, and the ability to generate overshooting APs), indicating that they represent a functionally viable subset of neurons.

For voltage‐clamp recordings of sEPSCs, the same internal solution was used, and sEPSCs were recorded in whole‐cell voltage‐clamp gap‐free acquisition mode using Clampex 11.2 software (Molecular Devices) and analyzed with the Mini Analysis Program (v6.08, Synaptosoft).

### 4.3 Cortical organoid microelectrode array (MEA) recordings

Seventy‐five‐day‐old organoids were plated onto 48‐well CytoViewMEA plates (Axion BioSystems, M768‐tMEA‐48B) coated with the same PLO‐Laminin solution described previously and maintained for the first week in neuronal maturation medium, followed by a change to the electrophysiology‐supporting medium. Network activity recordings were performed between days 120 and 140, with MEA plates stabilized for at least 5 min at 37°C and 5% CO_2_. Three‐minute recordings were analyzed with Axion's Neural Metrics Tool, version 2.4, with voltage spikes set to 5.5 standard deviations (SDs) above the background noise and active electrodes defined as more than 5 spikes/min. Wells with <25% active electrodes were excluded. Parameters measured included mean firing rate, weighted mean firing weight, and bursting frequency.^8^

### 4.4 Preparation and immunostaining of cortical organoid slices

For immunostaining experiments, organoids were collected on Day 30 and Day 120. Organoids were fixed with 4% paraformaldehyde (PFA) overnight at 4°C, and then dehydrated in a 30% sucrose solution in Dulbecco's Phosphate‐Buffered Saline (DPBS) for 3–4 days. Samples were embedded in O.C.T. Compound (Sakura Finetek USA) and stored at −80°C. Cryostat sections were cut at 20 μm thickness for SYN1/PSD95 quantification and 40 μm for neural rosettes and cortical markers.

For immunostaining, sections were air‐dried, washed three times with DPBS, and outlined with a hydrophobic marker. Blocking was performed with 0.3% Triton X and 5% BSA in PBS for 1 h at room temperature. Sections were incubated overnight at 4°C with primary antibodies in 0.1% Triton X and 1% BSA in PBS. Primary antibodies included: chicken anti‐MAP2 (microtubule‐associated protein‐2, Novus biologicals, Catalog No. NB300‐213, 1:1000), rabbit‐anti SOX2 (SRY‐Box Transcription Factor 2, Cell Signaling Technology, Catalog No. D6D9, 1:500), mouse anti‐Ki67 (Human Ki67 Pure B56, BD Biosciences, Catalog No. 556003, 1:200), mouse anti‐NEUN (NeuN, Catalog No. E4M5P, 1:500), mouse anti‐Synapsin1 (SYN1, Synaptic Systems, Catalog No. 106011, 1:200), rabbit anti‐PSD95 (Invitrogen, Catalog No. PIPA585749, 1:200), and Rabbit Anti‐Cleaved Caspase‐3 (Asp175) Antibody (Cell Signaling, Catalog No. 9661). The next day, sections were washed with PBS three times and incubated with secondary antibodies (Invitrogen, 1:500) for 2 h at room temperature in the dark. Sections were washed three times with DPBS, and DAPI counterstain was applied either with the VECTASHIELD mounting medium or during secondary antibody incubation.

### 4.5 Quantification of SYN1/PSD95 in cortical organoids

Synaptic proteins SYN1 and PSD95 were quantified along the MAP2‐positive area, which indicates the area occupied by the organoid slice. Images were acquired using a Zeiss LSM 900 Confocal Microscope with a 63X oil immersion objective. Laser settings and field of view size were maintained consistently throughout the imaging process. For analysis, .ics images were analyzed using an automated script from Zen Blue (Zeiss). At least three slices per organoid were selected, with at least three images per slice used for quantifications.

### 4.6 hiPSC‐derived cortical organoid bulk RNA‐sequencing data

Cortical organoids between 130 and 150 days were centrifuged at 300 x *g* for 5 min, washed once with PBS, and resuspended in ML lysis buffer (Macherey‐Nagel, Catalog No. 740971.50). One organoid was collected per tube and stored at −80°C before RNA Extraction. Total RNA was extracted from the cortical organoids using the NucleoSpin miRNA Kit for the Isolation of Small and Large RNA (Macherey‐Nagel, Catalog No. 740971.50) according to the manufacturer's instructions. For cDNA amplification, reverse transcription was performed using the Maxima First Strand cDNA Synthesis Kit and sent to Eurofins Genomics (eurofins.com) for genotype confirmation. Samples were sent to the Purdue University Genomics core at the Bindley Bioscience Center for quality control, library preparation, and sequencing. RNA quality was assessed via Qubit fluorimetry and Agilent TapeStation. Only samples with RNA integrity number (RIN) values greater than 8.8 were included in the analysis.

Raw datasets from the Element Biosciences AVITI sequencer. Reads were trimmed using fastp (v0.23.2), Trimmed reads were aligned to the primary assembly reference of the Homo sapiens genome (GRCh38) from Ensembl release 104 using STAR Aligner version (v2.7.10b)^30^ in two‐pass mode. Read alignments were assigned to genes using featureCounts (v2.0.1)^31^ in paired‐end mode and quantified on the reverse strand.

Genes exhibiting at least five reads in more than two samples were selected for upper‐quartile normalization. For differential expression analysis, WT samples were designated as the control and L1342P samples were designated as the experimental group. Generalized liner model (GLM) regression using RUVSeq (version 1.28.0, RUVr approach)^32^ was used to generate residuals based on the covariates of interest and incorporating them into the design matrix in edgeR (version 3.36.0),^33^ using R (version 4.1.3).^34^ Subsequently, edgeR was employed to fit a quasi‐likelihood negative binomial generalized log‐linear model and conduct gene‐wise statistical tests. Genes with a False Discovery Rate (FDR) of less than 0.05 were classified as differentially expressed.

For pathway analyses, the clusterProfiler package (version 4.10.0, R version 4.3.2)^34^, ^35^ and the org.Hs.eg.db package (version 3.18.0) were used to perform over‐representation analyses, utilizing the filtered genes from above as the background, with a FDR control at 5%. Enrichment analyses for Gene Ontology (GO) terms and KEGG/Reactome pathways were conducted separately for differentially expressed genes. The enrichplot (version 1.23.1.992) package was used to visualize the enrichment results and plotted in GraphPad Prism (version 9.5.1). The Volcano plot was generated using ggVolcanoPlot (https://ggvolcanor.erc.monash.edu).^36^

### 4.7 Statistical analysis

Statistical analysis was performed with GraphPad Prism (version 9.5.1). Depending on the dataset, tests included unpaired Student's *t*‐test, Mann–Whitney *U* tests, or one‐way analysis of variance (ANOVA). Results were presented as mean ± standard error of the mean (SEM). Values are shown in figures as *p < 0.05, **p < 0.01, ***p < 0.001, ****p < 0.0001, and n.s. (not significant) as *p* ≥ 0.05. Detailed statistical methods were outlined in the figure legends.

## AUTHOR CONTRIBUTIONS

M.I.O.A. and Y.Y. designed research. W.C.S. contributed unpublished reagents/analytic tools. M.I.O.A., M.R., Z.Q., K.V.S.S., Z.Z., H.H., V.S., S.H., M.W., C.O., H.Ka., N.A.L., M.H., C.D., T.N., K.W., B.Z., L.Y., N.C., X.C., J.Z., J.W., B.D., and Y.Z. performed research. M.I.O.A., M.R., K.V.S.S., V.S., M.W., M.O., H.H., T.N., C.Y., H.Ko., N.A.L., F.G., and Y.Y. analyzed data. M.A. provided the clinical description of the patient. M.I.O.A., M.R., and Y.Y. wrote the article.

## FUNDING INFORMATION

This work was supported by National Institutes of Health National Institute of Neurological Disorders and Stroke (NINDS) Grants R01 NS117585 and R01 NS123154 (to Y.Y.). M.I.O.A. is supported by the Fulbright‐Colciencias Scholarship Program. Support was also provided by the Purdue Institute for Cancer Research (PICR, Grant P30CA023168) and the Borch Department of Medicinal Chemistry and Molecular Pharmacology. M.R. is supported by the American Epilepsy Society Postdoctoral Fellowship and the Purdue University Lillian Gilbreth Postdoctoral Engineering Fellowship. The Yang Lab thanks the *FamilieSCN2A* Foundation for the Action Potential and Hodgkin‐Huxley Award.

## CONFLICT OF INTEREST STATEMENT

The authors declare no conflicts of interest.

## ETHICAL PUBLICATION STATEMENT

We confirm that we have read the Journal's position on issues involved in ethical publication and affirm that this report is consistent with those guidelines.

## Supporting information


Figure S1.
Figure S2.

## Data Availability

The data that support the findings of this study are available from the corresponding author upon reasonable request.
