# Supplementary material for: Epilepsy‐associated SCN2A‐L1342P mutation drives network hyperexcitability and widespread transcriptomic changes in human cortical organoids
Source: Epilepsia. 2026 Jul 30;67(10):5514–30. doi: 10.1002/epi.70392 (PMC13675769; doi:10.1002/epi.70392)
Supplement: Supplementary file 1 [file EPI-67-5514-s001.docx]

**Supplementary Figures**

**Figure S1.** Pluripotency marker expression is maintained across edited hiPSC lines, and no off-target editing was detected at different sodium channel isoform (*SCN1A*, *SCN3A*, and *SCN8A*) off-target sites.

**(A-C)** Immunofluorescence staining of pluripotency markers in control and edited induced pluripotent stem cell (iPSC) lines. Cells were stained for DAPI (blue, nuclei), canonical pluripotency markers including SOX2, OCT4, NANOG (green), and surface markers TRA-1-60, TRA-1-81, and SSEA4 (magenta). Merged images are shown in the rightmost panels. **(A)** Control hiPSCs, **(B)** L1342P, and **(C)** revertant P1342L. All conditions exhibit comparable expression of pluripotency markers, indicating no observable loss or alteration of pluripotent identity following editing.

**(D)** Potential off-target sequences for the CRISPR-Cas9 guide used to generate the SCN2A-L1342P, showing mismatches (red) in related sodium channel genes (SCN1A, SCN3A, SCN8A).

**(E–F)** Sanger sequencing traces of the indicated sodium channel loci in hiPSC-derived cortical organoid lines: A03 WT, A12 HET, D08 REV, and A07 REV. The boxed region indicates the CRISPR spacer sequence. No sequence alterations were detected in the potential off-target loci across all lines, demonstrating the specificity of the genome editing.

**Figure S2. Nav1.2-L1342P cortical organoids exhibit increased apoptosis compared to WT control.**

**(A)** Representative immunofluorescence images of control (WT) and Nav1.2-L1342P cortical organoid sections stained for cleaved caspase-3 (CC3; green) and MAP2 (gray). Dashed lines outline the organoid edge.

**(B)** Quantification of CC3+ cells normalized to total cell number reveals a significant increase in apoptotic cells in Nav1.2-L1342P organoids compared to controls (Control: 2.36 ± 0.66, N = 12 organoids, n = 17 images, 3 clones; L1342P: 9.56 ± 2.04, N = 10 organoids, n = 21 images, 1 clones, **p = 0.0026, Welch’s t-test).

**(C)** Quantification of CC3+ area normalized to the total area occupied by the MAP2 stain number reveals a significant increase in apoptotic cells in Nav1.2-L1342P organoids compared to controls (Control: 7.67 ± 1.31, N = 12 organoids, n = 17 images, 3 clones; L1342P: 14.42 ± 2.22, N = 10 organoids, n = 21 images, 1 clones, *p = 0.0135, Welch’s t-test).
